# Application of Experimental Design in Preparation of Nanoliposomes Containing Hyaluronidase

**DOI:** 10.1155/2014/948650

**Published:** 2014-09-09

**Authors:** Narayanan Kasinathan, Subrahmanyam Mallikarjuna Volety, Venkata Rao Josyula

**Affiliations:** Department of Pharmaceutical Biotechnology, Manipal College of Pharmaceutical Sciences, Manipal University, Manipal 576 104, India

## Abstract

Hyaluronidase is an enzyme that catalyzes breakdown of hyaluronic acid. This property is utilized for hypodermoclysis and for treating extravasation injury. Hyaluronidase is further studied for possible application as an adjuvant for increasing the efficacy of other drugs. Development of suitable carrier system for hyaluronidase would help in coadministration of other drugs. In the present study, the hyaluronidase was encapsulated in liposomes. The effect of variables, namely, phosphatidylcholine (PC), cholesterol, temperature during film formation (*T*_1_), and speed of rotation of the flask during film formation (SPR) on percentage of protein encapsulation, was first analyzed using factorial design. The study showed that level of phosphatidylcholine had the maximum effect on the outcome. The effect of interaction of PC and SPR required for preparation of nanoliposomes was identified by central composite design (CCD). The dependent variables were percentage protein encapsulation, particle size, and zeta potential. The study showed that ideal conditions for production of hyaluronidase loaded nanoliposomes are PC—140 mg and cholesterol 1/5th of PC when the SPR is 150 rpm and *T*_1_ is 50°C.

## 1. Introduction

Hyaluronic acid (HA) is a polysaccharide containing alternating units of glucuronic acid and glucosamine [1]. HA is distributed in tissues and is particularly abundant in extracellular matrices. Apart from the cellular and molecular functions, HA protects local tissues and cells against compression. HA, because of its high swelling property and viscous nature, restricts the movement of molecules including pharmacological agents across the tissues [2]. Hyaluronidase is an enzyme that catalyzes breakdown of HA. Approved label use of hyaluronidase includes treatment of extravasation injury, for hypodermoclysis and urography. Of late, hyaluronidase is tested for management of secondary complications associated with plastic surgery [3] and as an adjunct in improving the efficacy of pharmacological agents [4].

Use of hyaluronidase as an adjuvant therapy for improving the pharmacokinetic properties of coadministered drug is of particular interest as many of the regulatory bodies including US FDA have approved its use in humans [5]. The potential use of hyaluronidase as an adjunct could be exploited if a suitable carrier based delivery system for hyaluronidase is developed. This would allow coadministration of a second drug by directly incorporating them in the same delivery system and administering both drugs as a single dosage form. Few studies demonstrated that the efficacy of anticancer drug [6] and local anesthetics [4, 7] could be improved by pretreating the target site with hyaluronidase.

Understanding the effect of variables on the outcome is important for developing a product with all the predetermined requirements [8]. This will also ensure that such products do not show any batch to batch variation [9]. The effect of process variables can be studied either by checking the effect of one independent variable on the dependent variable at a given time or by using statistical tools, namely, experimental design. In experimental design, all the independent variables are varied together such that the effect of their interaction on the dependent variable is also analyzed [10]. The recent regulatory requirement suggests that quality by design approach should be adopted to build “quality into the product” [11, 12]. This can be achieved by defining and analyzing the effect of variables on the outcome using experimental design. The product developed using these statistical tools shows more consistency in terms of quality and reproducibility [13]. Experimental design using statistical tools such as factorial design and response surface methodology allows the researchers to rapidly study and optimize the conditions to achieve products with the best possible quality and attributes using the limited available resources and without losing any essential information that may affect the outcome [10, 14].

Liposomal delivery of proteins is one of the widely explored methods for delivery of proteins. In the present study, hyaluronidase was encapsulated in liposomes. The effect of process variables on size, percentage drug encapsulation, and zeta potential was studied using factorial design and CCD.

## 2. Materials and Methods

### 2.1. Chemicals

Cholesterol (extra pure) and chloroform (HPLC grade) were purchased from SRL Pvt. Ltd., (India) and Merck (India), respectively. L-*α*-phosphatidylcholine and triton-X 100 were obtained from Sigma (India). Lyophilized hyaluronidase (Hynidase Injection I.P. (Ovine), marketed by Shreya Life Sciences Pvt. Ltd., India) was used as drug source.

### 2.2. Methods

#### 2.2.1. Preparation of Liposomes

Hyaluronidase loaded liposomes were prepared using thin film hydration method. Lipids (Table 1 gives the amount of phosphatidylcholine (PC) added during the experiment) were dissolved in 20 mL of chloroform taken in a 250 mL capacity round bottom flask. The flask was rotated continuously (rpm as per the Table 1) as the solvent was removed by evaporation under vacuum at temperature (*T*_1_) indicated in Table 1 (Buchi Rotavapor R-215) and the lipids were allowed to deposit as a thin film within the flask. Traces of solvent (if any) were removed through desiccation. The lipid layer was hydrated with phosphate buffer saline (10 mL) (pH 7.4) containing hyaluronidase (protein content equivalent to 0.5 mg) in an orbital shaker (Remi Laboratory instruments, India, Model-CIS-24 BL) at 150 rpm, 28°C for overnight.

#### 2.2.2. Size Measurement

Liposomes formed after overnight hydration were sonicated at 60% amplitude control (probe sonicator, Sonics and materials Inc., USA) for total duration of 30 sec consisting of three cycles. Duration of each cycle was 10 sec with 10 sec interval. The formulation was maintained on ice bath during sonication. The liposomes were then transferred into polypropylene tubes and centrifuged at 450 g for 3 min to remove the coarse particles. The supernatant was then centrifuged at 34,600 g for 60 min (Sigma Laborzentrifugen 3k30, Germany) to collect the nanoparticles. The pellet obtained was used for calculating the particle size and percentage drug encapsulation. For size measurement pellet was dispersed in ultrapure water (Milli-Q, Millipore) and analyzed using photon correlation spectroscopy (Malvern Zetasizer). Nanoliposomes were made to release entrapped protein by treating them with 1% triton X-100 [15] and the amount of protein was estimated using Lowry method [16]. The amount of protein was calculated by comparing the absorbance (Biospec-1601, Shimadzu, Japan) with the standard curve plotted using bovine serum albumin.

#### 2.2.3. Experimental Design

The effect of variables involved in preparation of hyaluronidase loaded nanoliposomes was studied in two stages. First the effect of PC, cholesterol, temperature during film formation (solvent evaporation) (*T*_1_), and speed of rotation of the flask (rpm) during film formation (SPR) on percentage drug (hyaluronidase) encapsulation was analyzed using fractional factorial design. The design had eight runs with zero centre points and single base for the selected four factors. The effect of interaction of two variables showing the maximum effect on protein encapsulation was further studied for the effect on protein encapsulation, size, and zeta potential using CCD. The level of other variables was fixed based on the results of fractional factorial deign. CCD consisted of thirteen runs for the two selected factors. Alpha level was maintained at 1.414.

## 3. Results and Discussion

Factorial studies showed that the outcome, that is, percentage of protein encapsulated, was significantly affected by the amount of PC present in the system. One-way ANOVA showed that the effect of PC on percentage of drug encapsulation was highly significant with a *P* value less than 0.003. More than three-fourth of the outcome was contingent on the level of PC. The amount of the protein that could be entrapped in a liposome depends on the size of the protein and the amount of free aqueous phase within the vesicle. The portion of the aqueous phase interacting with lipid layer will be unavailable for the protein to occupy [17]. In the present study, the other independent variables did not have any significant effect on the outcome (*P* value > 0.05). Although cholesterol as an independent variable did not have significant effect on the outcome, two-way ANOVA showed that the interaction of PC with cholesterol has significant influence on the outcome. This is due to the steric stability that cholesterol provides by controlling the lipid layer fluidity [18]. Inclusion of cholesterol is mandatory particularly in case of hydrophilic drugs as cholesterol reduces the leakage of the entrapped drug besides improving the stability of liposomes [19, 20]. Interaction among the other independent variables did not have significant effect on the outcome (Table 1).

**Figure 1:**
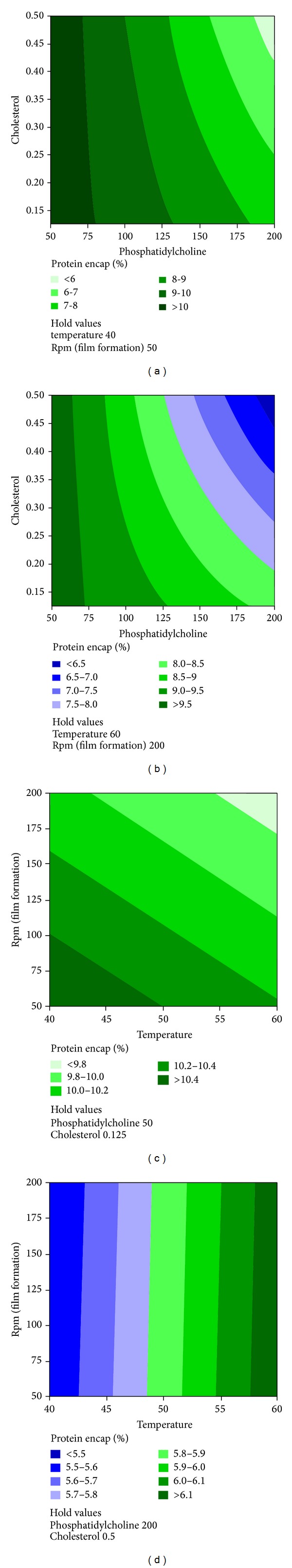
Contour plot for factorial studies. (a) The effect of various levels of phosphatidylcholine and cholesterol on percentage protein encapsulation when the level of the other variables was maintained at their lowest level. (b) The effect of various levels of phosphatidylcholine and cholesterol on percentage protein encapsulation when the level of the other variables was maintained at their highest level. (c) The effect of various levels of temperature (during film formation) and speed of rotation of the flask (rpm) on percentage protein encapsulation when the level of the other variables was maintained at their lowest level. (d) The effect of various levels of temperature (during film formation) and speed of rotation of the flask (rpm) on percentage protein encapsulation when the level of the other variables was maintained at their highest level.

**Figure 2:**
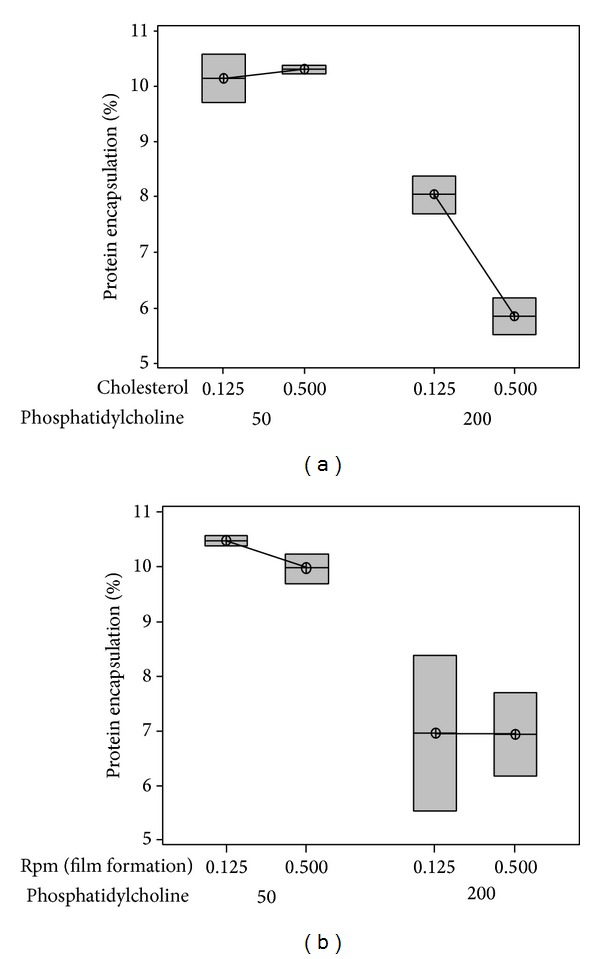
Box plot showing the effect of two-way interaction of independent variables on the outcome. (a) shows the effect of interaction of cholesterol and phosphatidylcholine on percentage protein encapsulation. (b) shows the effect of interaction of rpm and phosphatidylcholine on percentage protein encapsulation.

The outcome had very high dependence on the level of PC to an extent that the outcome was not affected by the level of other independent variables, namely, *T*_1_ and SPR. However, when the level of PC and cholesterol was maintained at their lowest levels, a higher amount of protein could be encapsulated if the SPR and *T*_1_ are maintained at a lower level (Figures 1(a) and 1(b)). When the level of lipids was high, the outcome, despite showing small dependence on *T*_1_, was unaffected by the SPR. However, the true effect of *T*_1_ was dependent on their interaction with SPR (Figures 1(c) and 1(d)). While *P* value of temperature and rpm were 0.933 and 0.886, their interaction had *P* value 0.536. This shows that, although individual effect of *T*_1_ and SPR and their interaction on outcome is insignificant, the effect of each other on the outcome depends on the level of interaction between them. The effect of two-way interaction of the independent variables on percentage drug encapsulation is given in Figures 2(a) and 2(b).

CCD study showed that PC had significant effect (*P* value < 0.05) on all the dependent variables, namely, percentage of protein encapsulation, particle size, and zeta potential (Table 2). However, the SPR had significant influence only on size and the other two dependent variables were not affected much by the SPR. The interaction between PC and SPR also had significant effect on size (Table 2).

Although both the independent variables (PC and SPR) could affect the outcome, SPR is a physical parameter whose effect is always going to depend on the level of PC. SPR affects the distribution and homogeneity of the film within the flask [21]. As the solvent is allowed to evaporate under vacuum, the lipids will be deposited as a film within the flask. However, the distribution of the film will be affected by the SPR [22]. As a result, the size of liposomes will be affected when the lipid layer is hydrated since a uniform and thin film will help in producing uniform small sized particles. However, entrapment was mainly dependent on the amount of PC. In w/o liposomes the amount of protein affected will depend on the amount of aqueous layer. Although aqueous phase will be sterically affected by the amount of lipid present in the lipid layer, an optimum level of PC (with cholesterol) is required to obtain stable liposomes without drug leakage.

**Figure 3:**
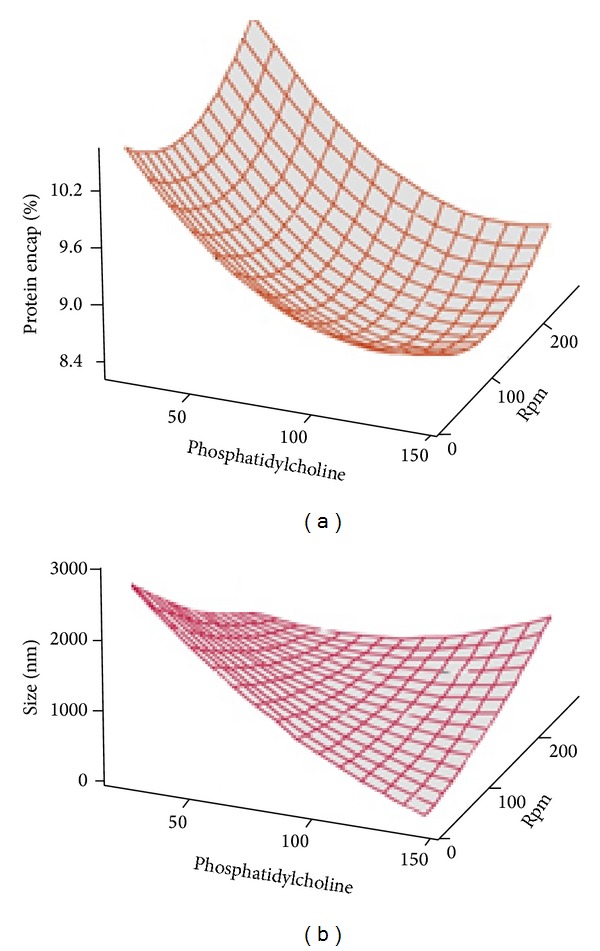
Surface plot for CCD studies showing the effect of interaction of phosphatidylcholine and speed of rotation of flask during film formation (rpm) on percentage of protein encapsulation (a) and size (b) when the level of other independent variables was maintained at a predetermined level.

**Figure 4:**
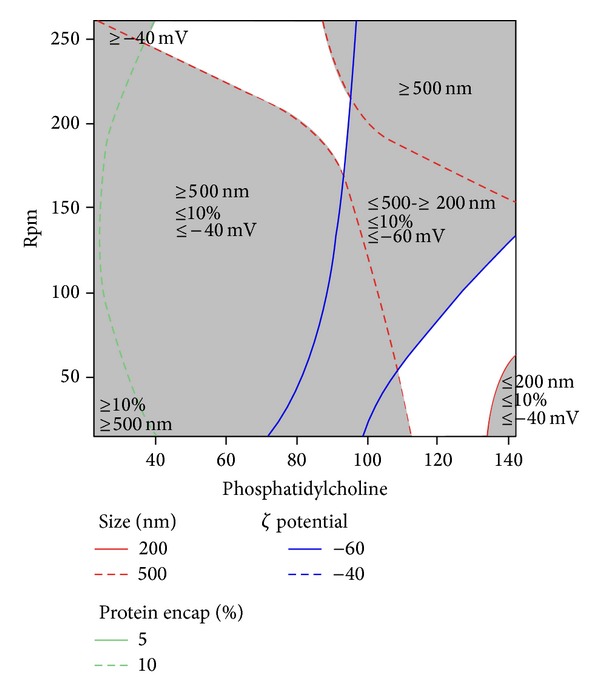
Overlaid contour plot showing the effect of interaction of phosphatidylcholine and speed of rotation of the flask (rpm) during film formation on size, percentage protein encapsulation, and zeta potential.

The percentage of protein encapsulated in all the trials during CCD was between 8 and 10% (Table 2). The levels of the independent variables were decided based on the outcome of factorial design (Table 1 and Figure 1). This could have contributed to the closeness in the values of the outcome. Surface plot (Figure 3(a)) showed that percentage of protein encapsulated could be improved by maintaining the level of PC at its lowest level and SPR either at lowest or highest level. However, particle size of less than 500 nm would be obtained only when the amount of PC is between 120 and 140 with SPR less than 150 rpm (Figure 3(b)). At these conditions, the zeta potential will be less than −50 mV (Figure 4). The effect of both the independent variables (PC and speed SPR) on all three dependent variables could be clearly observed from overlaid contour (Figure 4). Overlaid contour plot shows that a percentage protein encapsulation of above 8% could be obtained if the level PC is maintained above 100 mg and SPR below 150 rpm.

**Figure 5:**
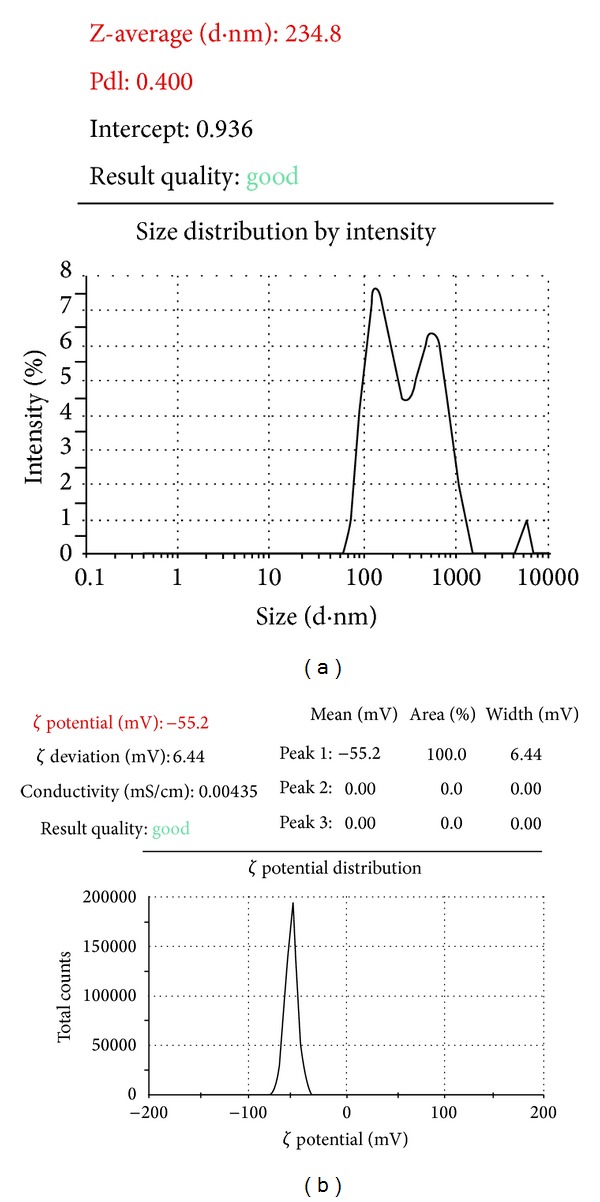
Size (a) and zeta potential (b) of hyaluronidase loaded nanoliposomes prepared using optimized conditions.

The study on the effect of independent variables on zeta potential showed that PC had significant influence on the outcome (Table 2). SPR had more influence on the outcome (zeta potential when the level of PC was maintained low (<45 mg)). When the level of PC was maintained high, SPR had less influence on the outcome. The study showed that good stable liposomes would be obtained when the level of PC is either between 40 and 80 mg or 110 and 140 mg with SPR as predicted in Figure 4 and excellent stability between 90 and 110 mg with SPR as per Figure 4. Size and zeta potential of hyaluronidase loaded nanoliposome are shown in Figure 5.

## 4. Conclusion

Experimental design was used to identify the main variables with significant effect and effect of interaction among the individual variables on the outcome in terms of drug encapsulation efficiency, mean particle size, and zeta potential of nanoliposomes containing hyaluronidase. Fractional factorial study showed that phosphatidylcholine was the critical component whose level determines the percentage of protein encapsulation. The ideal conditions for production of particles with least possible and maximum encapsulation efficiency and good zeta potential were studied and identified using CCD. Under the optimized conditions (phosphatidylcholine − 140 mg, cholesterol − 1/5th of phosphatidylcholine, temperature during film formation − 50°C, and speed of rotation of flask during film formation − 150 rpm) the mean particle size was found to be 245 ± 9 nm with percentage protein encapsulation of 10 ± 2% and zeta potential −53.7 ± 3.5 mV.

## Figures and Tables

**Table 1:** Fractional factorial design showing the effect of independent variables on the outcome.

Run order	PC (A) (%w/v)	Ch (B) (%w/v)	*T* (C) °C	SPR (D) rpm	% PE
1	50	0.5	40	200	10.22
2	200	0.125	40	200	7.69
3	200	0.5	40	50	5.51
4	50	0.125	60	200	9.7
5	200	0.125	60	50	8.38
6	50	0.5	60	50	10.38
7	200	0.5	60	200	6.17
8	50	0.125	40	50	10.58

Effect	−3.28	−1.02	0.16	−0.27	NA
	AB—1.18	AC—0.52	AD—0.25	NA	
% cont.	79.12	7.59	0.18	0.52	
	AB—10.18	AC—1.96	AD—0.46	NA	
*P* value					
OWA	0.003	0.51	0.921	0.87	
TWA	AB—0.022	AC—0.55	AD—0.78	NA	

PC: phosphatidylcholine; Ch: cholesterol; *T*: temperature during film formation (solvent evaporation); SPR: speed of rotation (during film formation); % PE: percentage of protein encapsulation; % cont.: percentage contribution.

OWA: one-way ANOVA; TWA: two-way ANOVA; AB, AC, and AD: interaction between phosphatidylcholine-cholesterol, phosphatidylcholine-temperature, and phosphatidylcholine-speed of rotation, respectively. NA: not applicable.

**Table 2:** Central composite design showing the effect of independent variables on the dependent variable.

Std. order	Run order	PC (A) (%w/v) (A)	SPR (rpm) (B)	% PE	Size (nm)	ZP (mV)
3	1	40	225	9.58	562.9	−49.2
5	2	22.40	137.5	10.02	1388	−42.9
7	3	82.5	13.76	9.00	837.8	−60.1
8	4	82.5	261.24	9.42	505.2	−54
6	5	142.60	137.5	8.60	273.8	−59.6
9	6	82.5	137.5	8.67	557.2	−64.3
10	7	82.5	137.5	8.93	417.8	−51.3
2	8	125	50	8.67	558.3	−58.6
12	9	82.5	137.5	8.49	1110	−65
1	10	40	50	9.93	1978	−56.4
4	11	125	225	8.27	737	−64.8
11	12	82.5	137.5	8.11	446.5	−63.3
13	13	82.5	137.5	8.96	514.3	−51.3

		PC (A)		0.005	0.006	0.032
*P* value		SPR (B)		0.902	0.06	0.57
		A ∗ B		0.961	0.02	0.276

PC: phosphatidyl choline; SPR: speed of rotation (during film formation); % PE: percentage of protein encapsulation; ZP: zeta potential.

## References

[1] Fraser JRE, Laurent TC, Laurent UBG (1997). Hyaluronan: its nature, distribution, functions and turnover. *Journal of Internal Medicine*.

[2] Girish KS, Kemparaju K (2007). The magic glue hyaluronan and its eraser hyaluronidase: a biological overview. *Life Sciences*.

[3] Bailey SH, Fagien S, Rohrich RJ (2014). Changing role of hyaluronidase in plastic surgery. *Plastic and Reconstructive Surgery*.

[4] Rüschen H, Adams L, Bunce C (2013). Use of hyaluronidase as an adjunct to local anaesthetic eye blocks (Protocol). *Cochrane Database of Systematic Reviews*.

[5] Kaur Chugh P, Roy V (2014). Biosimilars: current scientific and regulatory considerations. *Current Clinical Pharmacology*.

[6] Kohno N, Ohnuma T, Truog P (1994). Effects of hyaluronidase on doxorubicins penetration into squamous carcinoma multicellular tumor spheroids and its cell lethality. *Journal of Cancer Research and Clinical Oncology*.

[7] Clark LE, Mellette JR (1994). The use of hyaluronidase as an adjunct to surgical procedures. *The Journal of Dermatologic Surgery and Oncology*.

[8] Shariat S, Badiee A, Jaafari MR, Mortazavi SA (2014). Optimization of a method to prepare liposomes containing HER2/Neu-derived peptide as a vaccine delivery system for breast cancer. *Iranian Journal of Pharmaceutical Research*.

[9] Lionberger RA, Lee SL, Lee L, Raw A, Yu LX (2008). Quality by design: concepts for ANDAs. *The AAPS Journal*.

[10] Czitrom V (1999). One-factor-at-a-time versus designed experiments. *The American Statistician*.

[11] Guideline ICHHT

[12] Sangshetti JN, Deshpande M, Arote R, Zaheer Z, Shinde DB (2014). Quality by design approach: regulatory need. *Arabian Journal of Chemistry*.

[13] Telford JK (2007). A brief introduction to design of experiments. *Johns Hopkins APL Technical Digest*.

[14] Loveymi BD, Jelvehgari M, Zakeri-Milani P, Valizadeh H (2012). Statistical optimization of oral vancomycin-eudragit RS nanoparticles using response surface methodology. *Iranian Journal of Pharmaceutical Research*.

[15] Ramana LN, Sethuraman S, Ranga U, Krishnan UM (2010). Development of a liposomal nanodelivery system for nevirapine. *Journal of Biomedical Science*.

[16] Lowry OH, Rosebrough NJ, Farr AL, Randall RJ (1951). Protein measurement with the Folin phenol reagent. *The Journal of Biological Chemistry*.

[17] Adrian G, Huang L (1979). Entrapment of proteins in phosphatidylcholine vesicles. *Biochemistry*.

[18] Abreu AS, Castanheira EMS, Queiroz M-JRP, Ferreira PMT, Vale-Silva LA, Pinto E (2011). Nanoliposomes for encapsulation and delivery of the potential antitumoral methyl 6-methoxy-3-(4-methoxyphenyl)-1H-indole-2-carboxylate. *Nanoscale Research Letters*.

[19] Cagdas FM, Ertugral N, Bucak S, Atay NZ (2011). Effect of preparation method and cholesterol on drug encapsulation studies by phospholipid liposomes. *Pharmaceutical Development and Technology*.

[20] Haeri A, Alinaghian B, Daeihamed M, Dadashzadeh S (2014). Preparation and characterization of stable nanoliposomal formulation of fluoxetine as a potential adjuvant therapy for drug-resistant tumors. *Iranian Journal of Pharmaceutical Research*.

[21] Ning M, Gu Z, Pan H, Yu H, Xiao K (2005). Preparation and in vitro evaluation of liposomal/niosomal delivery systems for antifungal drug clotrimazole. *Indian Journal of Experimental Biology*.

[22] Bhatia A, Kumar R, Katare OP (2004). Tamoxifen in topical liposomes: development, characterization and in-vitro evaluation. *Journal of Pharmacy and Pharmaceutical Sciences*.

